# Dr. Thomas, in Answer to Mr. Fogo

**Published:** 1806-02

**Authors:** Robert Thomas

**Affiliations:** Guildford, Surry


### *To the Editors of the Medical and Physical Journal.*

Gentlemen,

HAVING lately met with a small tract, from the pen of Mr. Fogo, of Newcastle, on the opinions of ancient and modern physicians, including observations on those of Dr. Cullen on Amenorrhœa; of Dr. Saunders on Diseases of the Liver; of Dr. Beddoes on Scrophula; and of mine on Negro Cachexy; I beg leave, through the medium of your Journal, to offer a few remarks on those criticisms which more immediately concern me. But previous to my doing so, I would admonish Mr. Fogo, whenever he in future assumes the office of a critic, to adopt the language of civility and polished life, in preference to offensive reproach, which neither amends the person reproved, nor does any honor to the censor.

In the tract above alluded to, this gentleman has endeavoured to convince us that Amenorrhœa, Chlorosis, Dyspepsia, Scrophula, and Cachexia Africana, instead of being different diseases, are in reality one and the same, and and no other than chronic inflammation of the liver. This is jumbling disorders together in a very strange manner, and giving us to understand, that the numerical list of them, to which the human frame has been supposed to be liable, is smaller than we have been accustomed to look on it. To prove his position, Mr. Fogo has given a general table of the symptoms which attend on these diseases, for the purpose of comparison; but from which, as has very properly been observed by the Editors of the Medical and Chirurgical Review, (See No. LXI. p. 19.) any other person would irresistibly be led to draw the opposite conclusion, viz. that the diseases in question were altogether dissimilar.

Some men are however desirous of attracting the attention of the public, by advancing opinions which bear the face of singularity and originality, and I should conceive that Mr. Fogo was actuated by this view; for surely he cannot have the vanity to suppose that he possessed a sounder judgment than any of the ancient and modern physicians, and that he has seen through errors which they could not. We are told by him, that chronic hepatitis is as common and frequent as the tooth-ach, is as readily detected, and sometimes as easily cured. We are likewise informed, that a person can seldom walk the streets, or mix in a large company, without observing several faces, pale, green, livid, or yellow, whose owners are labouring under this very disease. Can the reader refrain from laughter? No doubt, he will regard this wonderful discovery in a proper light; and I am sure I may safely predict, that he will not think it deserving of a parliamentary remuneration.

To the jaundiced eye, every object is *said* to appear yellow; and drawing the inference, I should suspect that Mr. Fogo laboured under a diseased liver himself; that his gall does not pass off in the usual way, but is suffused in his system; or that it overflows, and so, to get rid of it, he dips in it his pen, and attacks with bitterness all those writers, who entertain sentiments which are different from his own. He is greatly dissatisfied with Dr. Cullen for looking on Chlorosis as a primary disease, and tells us that it is a mere imaginary one. In speaking of Dr. Beddoes, he sarcastically asks, whether a great writer may not be compared to a great talker? and condemns him for considering Scrophula as affecting the mesenteric glands, in any other view than as another variety of diseased liver; and with respect to myself, not a little harsh and rude censure censure is bestowed on me, because I have treated Cachexia Africana in my Modern Practice of Physic, as a disorder perfectly distinct from Chronic Hepatitis.

Dr. Trotter reported a case (I think) Gastrodynia, in a late number of your Journal, and he is attacked by Mr. Fogo for misnaming the disease ; and is told, that it was really one of chronic inflammation of the liver: another practitioner communicates a case of Phthisis through the same channel, and he likewise is corrected, and informed, that his patient laboured truly under chronic hepatitis. Why this carping at the opinion of others? and how is it that Mr. Fogo, seated at his writing desk, should pretend to have a more accurate knowledge of diseases than those very gentlemen under whose care they immediately fall? He has never, I presume, been in a tropical climate, and therefore cannot have seen a single case of negro cachexy, and still he pretends to know its real nature much better than I do, who during an extensive practice of many years in the West-Indies, had almost daily opportunities of being consulted respecting it. On this very subject, indeed, the Editors of the London Medical Review have paid me a compliment; for they observe, (see Vol. VIII. p. 104.) that I have given one of the most satisfactory descriptions of the negro cachexy, called in our colonies dirt-eating, with which they are acquainted. My sentiments of the disease in question, moreover, accord in most points with those of Dr. Chisolme, (see Vol. II. p. 171, of your Journal) and they do not differ, I believe, from those expressed by Dr. Hunt, in his Treatise on the Diseases of Jamaica. Was it necessary, I could bring forward many other valuable authorities to support my opinions, and confute those of Mr. Fogo.

In his tract he asserts, that I am staggered at finding women, men, and boys, alike subject to Cachexia Africana, and that I allow it may be Chlorosis in the females, but know not what it is owing to in men and boys. The quotation is very incorrect. My words are as follow: That in many respects the negro cachexy bears a great similarity to chlorosis, but they differ in the circumstance, that the latter affects only females, and principally about the age at which menstruation ought to take place, whereas the former affects males as well as females, and is often to be met with in children.—Surely two diseases may have a similarity in some of their symptoms, and yet be distinct. In this light do I view Chlorosis and Cachexia Africana, and and with respect to Chronic Hepatitis, the difference is very wide indeed.

Mr. Fogo expresses great astonishment that a professional M. D. should see the liver schirrhous, and never suspect that it possibly might have some share in producing the symptoms; nor never examine the state of the liver before death, when there were all the symptoms of obstructed bile, costiveness, clay-coloured stools, &c. knowing, or ought to have known, that diseases of the liver are very frequent in hot climates; and which oversight, he sarcastically adds, must give the reader an unfavourable opinion of the author of the Modern Practice of Physic.

My answer to these illiberal criticisms is, that I look on the morbid appearances which are to be observed in the liver on dissection, not as the primary or original disease, but as the consequence of its long duration. At first a morbid acidity prevails in the stomach, the appetite at length fails, digestion is impaired, a vitiated action takes place throughout the alimentary tube, unhealthy chyle is assimilated, the mesenteric glands are indurated, and the liver also becomes *at last* of an increased size and scirrhous hardness.—This last symptom I however assert, does not exist at the commencement of the negro cachexy. No hardness is to be felt in that viscus at first, it is not apparently enlarged, nor have I ever found, even in a single instance, the patient to complain of a pain in the liver extending up into the shoulder, one of the characteristic symptoms of both acute and chronic hepatitis.

During an extensive practice of nine years in the West-Indies, I certainly met with some cases of chronic inflammation of the liver, but I by no means found the disease occurring so frequently as the tooth-ach, as asserted by Mr. Fogo, neither did I so generally encounter such pale, green, livid, or yellow faces, as he speaks of.—Surely the people of Newcastle, which place is the seat of this gentleman's practice, must have truly deplorable countenances, if his remarks are well founded. I have further to observe, that in no case of true chronic hepatitis did I ever notice a propensity in the patient to eat chalk, and far less dirt, or has any other physician, I may safely venture to allege.

Because I have adopted the doctrines of Dr. Cullen with respect to chlorosis, Mr. Fogo is pleased to say that I seem to have sucked the same nurse who reared the professor's annotator.

annotator. This is indeed substituting low wit for argument. It is of little consequence who my nurse was; but I trust I imbibed in my early infancy liberal principles, and that my intercourse with mankind, and particularly with those of my own profession, although I may sometimes differ from them in opinion, has been perfectly consistent therewith. With respect to the rudiments of my medical knowledge, I beg leave to inform Mr. Fogo, that they were attained under the immediate instruction of that celebrated teacher, whose doctrines he derides, because, probably, he never had the opportunity of being taught by him. If he has enjoyed that advantage, but little benefit has accrued to him, for he has strangely jumbled diseases together in his tract.

Neither as a practical physician, nor as the author of the Modern Practice of Physic, am I under the least apprehension of suffering in repute by the disapprobation of a man who has established no fame by any literary production of his own, and who is only known by carping at the doctrines of others, holding a higher rank in the profession than himself. The publication above alluded to has stood the test of criticism, and has received the universal applause of all those, whose proper province it is to report the different works that issue from the press, of which the reader may be satisfied by looking over the short extracts inserted below.*

---

* "This is a judicious compilation of facts from the best writers, which may be perused with great advantage by students, because the different subjects are treated with brevity and perspicuity. The author has chiefly followed Dr. Cullen in his classification of diseases and in his text, but he is by no means a servile copyist, for he has abridged with judgment, has added modern opinions and discoveries, has frequently introduced the result of his own experience, and his performance thus becomes a useful compendium of the present state of medical practice."

Monthly Review for June, 1802, p. 183.

"The style of Dr. Thomas is clear and unaffected, and the arrangement of his work sensible and convenient. Books of this kind, when properly executed, containing the opinions of the best writers on the several diseases, abridged and placed together before the reader, may serve for occasional reference to refresh the memory, and save the trouble of turning over numerous volumes; they may be useful also to students, or to persons whose avocations do not permit them to consult more elaborate treatises."

British Critic, vol. xx. p. 397.

"We have read this work with great satisfaction, and think it a concise, but judicious abstract of the Practice of Medicine. The author's description of diseases is clear and characteristic, his remedies appropriate and well chosen. His description of the yellow fever is peculiarly accurate."

Critical Review for August, 1804.

It has also been highly approved of by many physicians of the first eminence, as being a concise, but accurate compendium of the present state of medical practice, well calculated to guide the young practitioner, and even occasionally to remind those of long standing on points which may have escaped their recollection.

No doubt there are some physicians among your numerous readers and correspondents, who, from a residence in the West Indies, may be well acquainted with the nature of the Cachexia Africana, and therefore I beg leave to request the favor of their sentiments on this disease, through the medium of the Medical Journal; if my own are erroneous, I am open to conviction.

I am, &c.

*Guildford, Surry,*

*Dec.* 28, 1805.

ROBERT THOMAS, M.D.

---

### *To the Editors of the Medical and Physical Journal.*

Gentlemen,

